# The non-structural protein 5A (NS5A) of hepatitis C virus interacts with the SH3 domain of human Bin1 using non-canonical binding sites

**DOI:** 10.1186/2047-783X-19-S1-S9

**Published:** 2014-06-19

**Authors:** Melanie Schwarten, Zsófia Sólyom, Sophie Feuerstein, Amine Aladağ, Silke Hoffmann, Dieter Willbold, Bernhard Brutscher

**Affiliations:** 1 Institut de Biologie Structurale, Université Grenoble 1, 38027 Grenoble, France; 2 Commissariat à l’Energie Atomique et aux Energies Alternatives (CEA), 38054 Grenoble, France; 3 Centre National de Recherche Scientifique (CNRS), 38400 Grenoble, France; 4 Institute of Complex Systems (ICS-6) Structural Biochemistry, Forschungszentrum Jülich, 52425 Jülich, Germany; 5 Institute for Physical Biology, Heinrich Heine University, 40225 Düsseldorf, Germany

## Background

The hepatitis C virus (HCV) is a major human pathogen that causes severe diseases such as chronic hepatitis, liver cirrhosis and finally hepatocellular carcinoma. Although no enzymatic activity could be attributed yet to the HCV non-structural protein 5A (NS5A), it is indispensable for viral replication and particle assembly. Furthermore, it is associated with a variety of cellular pathways, although their relevance for viral pathogenesis still has to be elucidated. To fulfil its function NS5A interacts with a large number of different proteins including both viral and human ones. NS5A is organized into three domains, which are connected via two low complexity sequences (LCS). The first domain is highly conserved among different HCV genotypes and forms a well-defined globular structure [1]. The domains 2 (D2) and 3 (D3) are less conserved and intrinsically disordered. Nonetheless, three segments in LCS-I and D2 show significant propensities to adopt α-helical structures as could be shown by nuclear magnetic resonance (NMR) chemical shift and ^15^N relaxation data [2]. The LCS-II connecting D2 and D3 contains two directly neighbored class II PxxP-motifs, which are important for interactions with Src homology 3 (SH3) domains. SH3 domains mediate protein-protein interactions, often via binding to polyproline II helices. Recent studies also revealed alternative binding mechanisms, mainly involving helical motifs and positively charged amino acid residues. The SH3 domain of the bridging integrator 1 (Bin1) is known to interact with NS5A not only via its PxxP-motifs, but also via two non-canonical binding sites, which will be further described here [3].

## Materials and methods

The Bin1 SH3 domain and the NS5A fragment containing residues 191 to 340 (NS5A(191–340)) were expressed recombinantly as GST-fusions and purified. NMR experiments were performed on Agilent VNMRS 600 MHz and 800 MHz spectrometers equipped with cryogenically cooled triple-resonance (HCN) probes with pulsed z-field gradients. NMR data were processed using NMRPipe and evaluated using CcpNMR. The binding interfaces of NS5A(191–340) and Bin1-SH3 were determined by mapping chemical shift changes upon addition of increasing amounts of Bin1-SH3 to NS5A(191–340). To determine Carbonyl and C^α^ chemical shifts BEST-TROSY HNCO and HNCA experiments were used [4]. Secondary structure propensities were calculated based on HN, N, Carbonyl and C^α^ secondary chemical shifts of NS5A(191–340) in the free and bound state.

## Results

To study the interaction of Bin1 SH3 domain with NS5A via non-canonical binding sites, we used an NS5A fragment lacking the high-affinity SH3 binding site (PxxP motif). Based on NMR chemical shift perturbation experiments we could identify two regions in NS5A(191-340) interacting with the Bin1 SH3 domain. Binding site 1 (B1) comprises NS5A residues 200 to 228 whereas binding site 2 (B2) includes residues 295 to 320. These two binding sites have been shown previously to contain regions that transiently adopt α-helical structures. Next, we examined how Bin1 SH3 binding influences the conformational sampling properties of NS5A. To determine the secondary structure propensities ^13^C secondary chemical shifts can be used as sensitive probes. The secondary structural propensity (SSP) score combines all measured chemical shifts into a single value. It provides a quantitative measure of the percentage of conformers in the structural ensemble with α-helical geometry (positive SSP score) or extended geometry (negative SSP score). Based on the SSP scores NS5A(191-340) has three regions with α-helical propensities of approximately 40% for H1 (residues 205–221) and H2 (residues 251–266) and approximately 50% for H3 (residues 292–306) in the free state. Binding of the Bin1 SH3 domain affects the helical segments H1 and H3, which are part of the interaction regions B1 and B2, respectively. Unexpectedly, the α-helical structure was not selected by the SH3 domain, but a disordered one, as the SSP scores in segment B1 dropped down to almost zero, while for B2, they are significantly reduced (although no ^13^C chemical shifts could be measured for residues 214–217 and 302–309 due to extensive line broadening).

Finally the binding interface of the SH3 domain was determined. Therefore amide chemical shift changes of Bin1 SH3 residues upon interaction with NS5A(191-340) were measured and mapped on the Bin1 SH3 structure. Similar to a canonical interaction partner NS5A(191-340) binds to the classical binding pockets of SH3 domains comprising the RT loop and N-Src loop as well as β-strands 3 and 4.

## Conclusions

Besides the well-established interaction of NS5A with SH3 domains via a PxxP-motif we could show that two additional regions in NS5A interact with the SH3 domain of human Bin1. These regions (B1 and B2) do not contain a canonical PxxP-motif, but are highly positively charged. Although NS5A(191-340) is intrinsically disordered, it has three segments with increased α-helical propensity. B1 and B2 correspond to two of these segments in the free state. Remarkably, upon interaction with the Bin1-SH3 domain the α-helical propensity decreases and a fuzzy complex is formed.
